# Parkinson’s progression prediction using machine learning and serum cytokines

**DOI:** 10.1038/s41531-019-0086-4

**Published:** 2019-07-25

**Authors:** Diba Ahmadi Rastegar, Nicholas Ho, Glenda M. Halliday, Nicolas Dzamko

**Affiliations:** ForeFront Dementia and Movement Disorders Laboratory, Brain and Mind Centre, Central Clinical School, Faculty of Medicine and Health, University of Sydney, Camperdown, NSW 2050 Australia

**Keywords:** Parkinson's disease, Predictive markers

## Abstract

The heterogeneous nature of Parkinson’s disease (PD) symptoms and variability in their progression complicates patient treatment and interpretation of clinical trials. Consequently, there is much interest in developing models that can predict PD progression. In this study we have used serum samples from a clinically well characterized longitudinally followed Michael J Fox Foundation cohort of PD patients with and without the common leucine-rich repeat kinase 2 (LRRK2) G2019S mutation. We have measured 27 inflammatory cytokines and chemokines in serum at baseline and after 1 year to investigate cytokine stability. We then used the baseline measurements in conjunction with machine learning models to predict longitudinal clinical outcomes after 2 years follow up. Using the normalized root mean square error (NRMSE) as a measure of performance, the best prediction models were for the motor symptom severity scales, with NRMSE of 0.1123 for the Hoehn and Yahr scale and 0.1193 for the unified Parkinson’s disease rating scale part three (UPDRS III). For each model, the top variables contributing to prediction were identified, with the chemokines macrophage inflammatory protein one alpha (MIP1α), and monocyte chemoattractant protein one (MCP1) making the biggest peripheral contribution to prediction of Hoehn and Yahr and UPDRS III, respectively. These results provide information on the longitudinal assessment of peripheral inflammatory cytokines in PD and give evidence that peripheral cytokines may have utility for aiding prediction of PD progression using machine learning models.

## Introduction

Parkinson’s disease (PD) is a progressive neurodegenerative disorder that causes both motor and non-motor symptoms. The motor symptoms of PD, which include tremor, rigidity, postural instability, and bradykinesia, are a direct result of insufficient dopamine signaling due to the selective degeneration of dopamine producing neurons in the substantia nigra region of the midbrain. In addition, the more diverse and heterogeneous non-motor symptoms, which include autonomic dysfunction, hyposmia, cognitive decline, depression, and sleep dysfunction, appear to also be linked to the pathological accumulation of the α-synuclein protein.^1,2^ The exact causes of PD remain unclear but are thought to involve a complex interplay between genetics, biology, and the environment, which contributes to the underlying heterogeneity of not only PD symptoms, but also their rates of progression over time.^3^ This provides uncertainty to patients in regards to future quality of life, and also presents challenges for therapeutic trials in terms of defining successful endpoints.^4^

Altered peripheral immune system function is increasingly being reported for patients with PD. Indeed, evidence implicating α-synuclein pathology, the enteric nervous system, and the gut–brain axis in the etiology of PD suggests that PD may even commence in the periphery.^5–7^ This is intriguing and may provide an opportunity to identify peripheral markers that contribute to the prediction of PD progression. Inoculation of α-synuclein into the periphery can induce neuropathology in rodents,^7,8^ and notwithstanding the challenges of its detection, pathological α-synuclein has been identified in both intestinal and skin biopsies from prodromal PD cohorts.^9–12^ Pathological forms of α-synuclein have also been shown to directly modulate inflammatory pathways,^13–16^ and a number of studies have demonstrated a chronic low grade inflammatory phenotype in PD patients (for reviews see refs. ^17–20^). The exact mechanism by which this phenotype manifests remains to be determined, but as well as activation of inflammatory pathways, PD patients have also been reported to have altered populations of peripheral immune cells,^21–24^ and altered immune cell responses to activating stimuli,^25^ which collectively implicate immune dysfunction in PD.

In addition, many of the genes implicated in PD risk are also highly expressed in immune cells, with many being directly implicated in modulation of inflammatory signaling pathways.^20^ One such example is leucine-rich repeat kinase 2 (LRRK2), which is highly expressed in monocytes,^26,27^ and has been implicated in monocyte maturation and innate immune inflammatory pathways.^28–32^ At least six confirmed pathogenic missense mutations occur in LRRK2 and all serve to increase the enzyme’s catalytic kinase activity.^33,34^ The most common LRRK2 mutation is G2019S, which is the biggest cause of dominantly inherited PD and accounts for ~1–5% of all PD.^35^ We have previously shown that certain peripheral inflammatory cytokines are increased in a subset of asymptomatic mutation carriers with the LRRK2 G2019S mutations.^36^ Any extent to which peripheral inflammatory cytokines contribute to PD causality remains unclear, however, importantly, LRRK2 kinase inhibitors are in advanced stages of development^37^ and there is much interest in how clinical trials can be taken forward.

The heterogeneity of PD progression combined with a lack of objective biomarkers^38^ has however complicated both clinical trial design and outcomes in PD, and thus the need for better models of PD progression and/or better strategies for selection of participants into specific clinical trials is evident. The emergence of large datasets containing multimodal data from longitudinally followed cohorts such as the Michael J Fox Foundation Parkinson’s Progression Markers Initiative has facilitated novel machine learning approaches to develop predication models of PD progression.^39,40^ In the current study, we have employed machine learning algorithms to further explore the association between peripheral inflammatory cytokines and clinical PD symptomology using longitudinally collected serum samples from PD patients with and without the LRRK2 G2019S mutation. We provide initial evidence that peripheral inflammatory cytokines measured at baseline contribute to the prediction of longitudinal clinical outcomes. Our results provide new information on the longitudinal assessment of peripheral inflammatory cytokines and act as a starting point to develop more refined prediction models that may have utility in improving patient stratification and/or endpoint readouts for PD clinical trials.

## Results

### Evaluation of baseline clinical and cytokine data

**Fig. 1:**
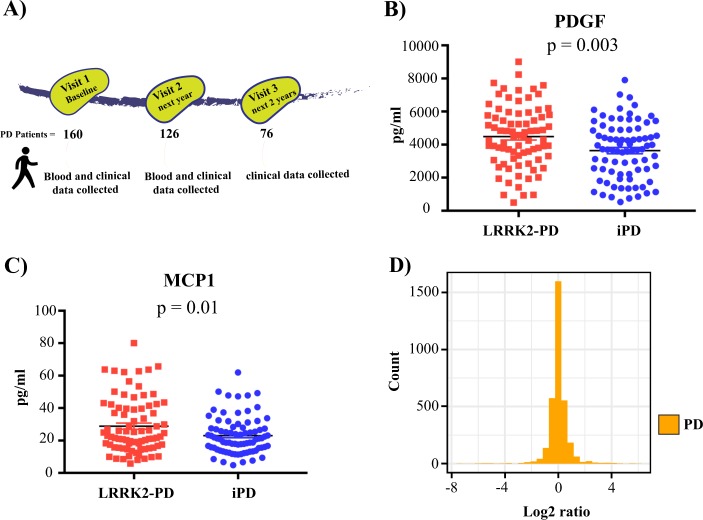
Increased levels of platelet-derived growth factor in LRRK2-PD serum. **a** Overview of patient sample numbers and data collection. Baseline platelet-derived growth factor **b** and monocyte chemoattractant protein 1 **c** levels in Parkinson’s disease patients with (red squares) and without (blue circles) the LRRK2 G2019S mutation. Data were compared using Student’s *t* test. Graphs show mean ± SEM, *n* = 80 per group. **d** Log2 changes over 1 year for all 27 cytokine measurements in all patients. *N* = 160

Our study utilized the Michael J Fox Foundation LRRK2 clinical consortium longitudinal sample collection. From this collection, we obtained serum samples and clinical data from 160 patients for a baseline comparison of LRRK2-PD and idiopathic PD (Fig. 1a). Age and gender were taken into consideration during sample selection and were thus well matched between the idiopathic and LRRK2-PD groups (Table 1). Age at diagnosis and the majority of clinical scores were also similar between the two groups. LRRK2-PD only significantly differed from the idiopathic group in regard to motor scores evaluated with the unified PD rating scale part 3 (UPDRSIII), and the University of Pennsylvania smell identification test (UPSIT) (Table 1). The lower UPDRSIII score for LRRK2-PD indicates milder motor disease, whilst the higher UPSIT score indicates less severe hyposmia in the LRRK2-PD group. As for the clinical scores, the majority of the assayed serum cytokines were similar between the two PD groups (Supplementary Table 1). Two exceptions were platelet-derived growth factor (PDGF) and monocyte chemoattractant protein one (MCP1), which were increased by 23% (*p* = 0.003) (Fig. 1b) and 27% (*p* = 0.01) (Fig. 1c), respectively, in the LRRK2-PD group. Of these two cytokines, PDGF remained significantly elevated in the LRRK2-PD group following univariate analysis covarying for age and gender (26% increase, *p* = 0.001), and after employing a Bonferroni correction for multiple comparisons. Levels of PDGF did not significantly correlate with any of the baseline clinical symptomology rating scales (Supplementary Table 2).

**Table 1:** Baseline demographic data

	Idiopathic PD	LRRK2 PD	Range at BL
*n*	80	80	–
Age (years)	68 ± 1	69 ± 1	–
Age at diagnosis	58 ± 1	57 ± 1	–
Gender (M/F)	54/26	40/40	–
Epworth sleep scale	8.0 ± 0.7	8.2 ± 0.6	2–21
Geriatric depression scale	3.9 ± 0.5	4.3 ± 0.5	0–14
Hoehn and Yahr	2.5 ± 0.1	2.3 ± 0.1	1–4
Schwab and England ADL	76.3 ± 3.0	77.8 ± 2.6	20–100
SCOPA-AUT	21.2 ± 1.7	21.3 ± 1.8	2–62
REM-sleep dysfunction	4.1 ± 0.5	3.1 ± 0.4	0–12
MoCA	25.0 ± 0.6	25.2 ± 0.5	10–30
UPSIT	11.5 ± 1.3	19.7 ± 1.5*	0–39
UPDRS III	24.8 ± 1.8	19.3 ± 1.6*	2–46

Baseline demographic for selected serum samples. Data are mean ± SEM.

*MoCA* Montreal cognitive assessment, *UPSIT*  University of Pennsylvania smell identification test, *ADL* activities of daily living, *UPDRS* *III* unified Parkinson’s disease rating scale part 3

*p < 0.05

### Evaluation of longitudinal data

In order to evaluate changes over a 1-year time period, cytokines were also measured for 126 of the PD patients for which longitudinal serum samples were available. Plots of log2 ratios of the change in cytokine measurements between the time points for each patient were generated. When all cytokines for both groups were considered together, ~90% of the measures changed either up or down by onefold or less over the 1-year period (Fig. 1d). Temporal changes for individual cytokines for the separate idiopathic and LRRK2-PD groups are shown in Supplementary Fig. 1, with the largest variation seen for granulocyte colony stimulating factor (GCSF) and interleukin (IL) five (IL-5). With the single exception of IL one receptor antagonist (IL-1RA) (*p* = 0.04), Kolmogorov–Smirnov tests showed no significant differences between the log2 distributions of the individual cytokines for the idiopathic and LRRK2-PD groups. We also determined the extent to which the different clinical variables changed over time, again with both the idiopathic and LRRK2-PD groups combined together. Longitudinal assessment of clinical data from the same individuals at baseline and with 1- and 2-year follow ups showed a significant increase in scores for the geriatric depression, Hoehn and Yahr and UPDRS III scales (Table 2). Increases in depression and motor symptom severity were associated with a significant decrease in the Schwab and England activities of daily living scale (Table 2). Symptoms predominantly associated with early PD including, olfaction, autonomic function, and sleep dysfunction were not significantly changed over the 3-year time course.

**Table 2:** Longitudinal progression of Parkinson’s disease symptomology

Clinical variables	Visit 1 baseline	Visit 2 + 1 year	Visit 3 + 2 years	*p*-value
Epworth sleep scale	8.789 ± 0.53	8.447 ± 0.57	8.75 ± 0.57	0.7848
Geriatric depression scale	3.167 ± 0.44	4.183 ± 0.46	4.167 ± 0.48	0.0025*
Hoehn and Yahr	2.31 ± 0.07	2.55 ± 0.10	2.62 ± 0.11	0.0005*
Schwab and England ADL	82.4 ± 1.84	78 ± 2.28	75.2 ± 2.52	0.0010*
SCOPA-AUT	22.12 ± 1.40	21.57 ± 1.55	22.36 ± 1.51	0.8528
REM SLEEP dysfunction	4.026 ± 0.37	3.671 ± 0.40	3.526 ± 0.37	0.2843
MoCA	26.46 ± 0.37	26.02 ± 0.42	25.7 ± 0.45	0.0903
UPSIT	16.89 ± 1.25	15.38 ± 1.23	15.88 ± 1.18	0.3351
UPDRS III	20.93 ± 1.26	22.05 ± 1.45	23.91 ± 1.59	0.0178*

Longitudinal Parkinson’s disease symptomology data from clinical rating scales with both LRRK2-PD and iPD combined. Data are mean ± SEM. *indicates *p* < 0.05 using repeated measures one-way analysis of variance, or in the case of the categorical Hoehn and Yahr scale, Kruskal–Wallis

*MoCA* Montreal cognitive assessment, *UPSIT* University of Pennsylvania smell identification test, *ADL* activities of daily living, *UPDRS* *III* unified Parkinson’s disease rating scale part 3

### Correlations between baseline cytokines and disease progression

We next determined if baseline cytokine levels were associated with changes in motor severity, depression, or the activities of daily living scale over time. Correlation analysis identified 14 cytokines that positively correlated significantly with the fold change in geriatric depression scale over the 2-year time period (Table 3). Seven cytokines also positively correlated significantly with the fold change in UPDRS III (Table 3), whilst no significant correlations were observed for the other two clinical scales assessed.

**Table 3:** Correlations between baseline cytokines and Parkinson’s disease symptomology

Cytokines	Geriatric depression scale	Cytokines	UPDRS III
IL5	0.609**	IL5	0.358**
IL8	0.415**	IL8	0.241*
GCSF	0.438**	GCSF	0.283*
MIP1β	0.294*	MCP1	0.237*
IL10	0.435**	IL10	0.315**
MIP1β	0.294*	IFNγ	0.401**
IL6	0.320*	IL15	0.247*
IL1RA	0.546**		
TNFα	0.436**		
CCL5	0.337*		
bFGF	0.301*		
VEGF	0.640**		
MIP1α	0.435**		
IL7	0.350*		
IL15	0.643**		

Correlation analysis was performed to identify any significant correlations between the log change in clinical scales over 3 years and baseline cytokine levels. The table shows the Pearson correlation coefficient with *p < 0.05 and **p < 0.01. *n* = 65

*UPDRS* *III* unified Parkinson’s disease rating scale part 3

### Machine learning predicting of longitudinal clinical outcomes

**Fig. 2:**
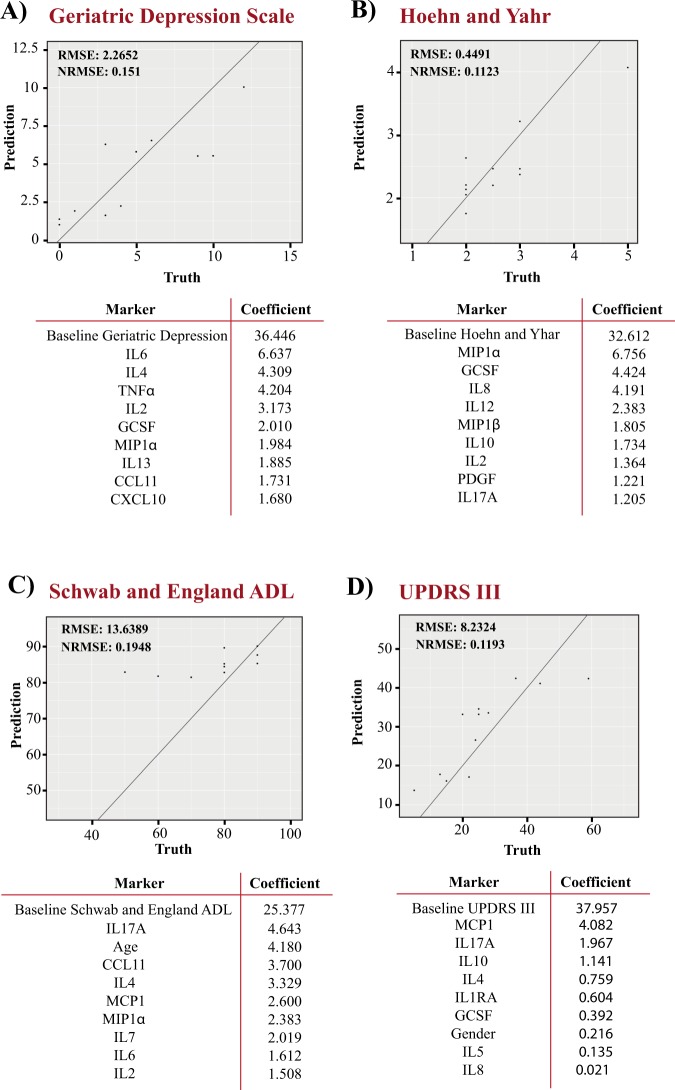
Random forest prediction of longitudinal clinical outcomes. Random forest machine learning algorithms using baseline cytokine measures were used to predict the 2-year outcomes for the geriatric depression scale **a**, Hoehn and Yahr **b**, Schwab and England activities of daily living **c**, and UPDRS III **d**. The root mean square error (RMSE) and normalized RMSE (NRMSE) are shown as an indication of performance for each predictive algorithm. For each algorithm the variables that contributed the most to the predictive performance are listed. Data points indicate the machine learning prediction for each individual in the test dataset. The lower the NRMSE value and the more the test data points lie along the prediction line, the better the prediction accuracy

To more formally quantify the extent that baseline peripheral cytokines contribute to the prediction of longitudinal clinical outcomes we employed machine learning. For the four clinical variables that showed significant changes over time, 80% of the samples for which longitudinal data were available were used to train elastic-net and random forest algorithms. Any individuals for which cytokine changes over 1 year were >2 SD from baseline were excluded, as such a dramatic change in cytokines likely indicates an underlying inflammatory condition. The final hyperparameters used and the performance of each model on training data are shown in Supplementary Table 3. Using normalized route mean square error (NRMSE) as a comparative measure of performance, the random forest model was better than elastic net for predicting all measured clinical outcomes (Supplementary Table 3). The optimized random forest models were therefore tested for their ability to predict the 2-year follow up measures for geriatric depression scale (Fig. 2a), Hoehn and Yahr (Fig. 2b), Schwab and England activities of daily living (Fig. 2c), and UPDRS III (Fig. 2d) using the withheld test dataset. NRMSE was used as a comparative measure of predictive performance, with the lower the value the more accurate the predictive ability of the model. NRMSE was best for predicting motor symptomology using either the Hoehn and Yahr scale, with NRMSE of 0.1123, or UPDRS III, with NRMSE of 0.1193 (Fig. 2). The use of random forest to predict longitudinal outcomes with the available baseline clinical variables, gave worse performance than using baseline cytokines (average 16% decrease in test NRMSE) (Supplementary Table 4). When clinical data and cytokines were combined for analysis, the performance of the random forest model was again reduced compared to using baseline cytokines alone (average 11% decrease in test NRMSE) (Supplementary Table 4).

### Relative contribution of peripheral cytokines to prediction models

The top ten variables contributing to each predictive algorithm are also shown (Fig. 2). In all cases, the baseline value for each particular clinical measure was the main contributor to the predictive models. This likely reflects the subtle symptomology progression over 2 years in a cohort with PD already established for ~10 years. The main contributing cytokines for Hoehn and Yahr and UPDRS III were macrophage inflammatory protein one alpha (MIP1α) and MCP1, respectively. The cytokines GCSF, IL8, IL17A, and IL10 were amongst the top ten variables for both motor severity scale prediction models. IL-6 and IL-4 were the main contributing cytokines for prediction of the geriatric depression scale, and all the top cytokine variables also contributed to the prediction of the Schwab and England ADL scale. The demographic variables age and gender were among the top 10 contributors to the prediction of activities of daily living and UPDRS III scales, respectively (Fig. 2). LRRK2 mutation status was also included in the variables used for prediction but did not rank amongst the top variables for any prediction model.

## Discussion

In the current study we have longitudinally assessed peripheral inflammatory cytokines and employed machine learning algorithms to determine the relative extent to which the cytokines contribute to the longitudinal prediction of PD symptomology. We employed samples from the Michael J Fox Foundation LRRK2 clinical cohort consortium. This consortium has two sample collections, a multiethnic cross-sectional cohort that we have previously used to demonstrate increased peripheral inflammation in asymptomatic LRRK2 G2019S mutation carriers,^36^ and the longitudinally followed Ashkenazi Jewish cohort that we have used this time. Consistent with our previous results, we found that levels of inflammatory cytokines were largely similar between PD patients with and without the LRRK2 G2019S mutation. The exception was PDGF, which was increased in LRRK2 G2019S patients compared to idiopathic PD, again replicating our previous findings.^36^ That PDGF is elevated in LRRK2 G2019S patients compared to idiopathic PD in two independent cohorts suggests a role for the LRRK2 enzyme in regulating this cytokine.

One important aim of the current study was to evaluate the variability of peripheral inflammatory cytokine levels in individuals over a 1-year time frame. A number of factors may contribute to the regulation of peripheral cytokine levels,^41^ and whilst longitudinal studies have been conducted for some neurodegenerative diseases, such as Alzheimer’s disease,^42^ longitudinal assessment of peripheral inflammatory cytokines in PD is lacking. At a group level we found variability for the majority of cytokine measures at both time points. However, on an individual level, cytokine levels were similar at the two time points for individuals in both the LRRK2 and idiopathic PD groups. That instances of higher inflammation were largely stable over the 1-year time frame adds to the utility of peripheral cytokines as PD biomarkers, however it would be important in future studies to further control for variables that can impact on inflammation including diet, recent illness, and/or other potential underlying inflammatory conditions.

Another major aim of the current study was to evaluate machine learning approaches for the prediction of clinical outcomes in PD using demographic and peripheral cytokine measures. Machine learning approaches may complement other statistical approaches used to model PD progression^43^ and can leverage extensive datasets whilst providing an unbiased quantitative measure of predictive performance. Previous studies have employed such approaches and used clinical, genetic, imaging, and cerebrospinal fluid markers to predict the initiation of symptomatic therapy^40^ and motor progression based on UPDRSIII.^39^ In the current study, we used peripheral cytokine measurements in combination with elastic net and random forest models to predict longitudinal clinical outcomes of depression, motor severity, and the activities of daily living scale. Of these clinical symptoms, random forest models of motor severity showed the best predictive performance using the baseline cytokine measurements. The use of cytokines provided an average 20% improvement in predictive ability over using clinical data alone. The cytokines that contributed the most to the predictive models of motor severity were MIP1α and MCP1 for the Hoehn and Yahr and UPDRS III scales, respectively. Both basal and lipopolysaccharide stimulated levels of MIP1α and MCP1 have previously been reported to be higher in PD patient peripheral mononuclear cells compared to controls.^44^ In our study, baseline levels of MCP1 were also positively correlated with longitudinal changes in UPDRS III, suggesting that higher levels of MCP1 are associated with faster motor progression. IL-8, IL-10, and GCSF were other cytokines that positively correlated with changes in UPDRS III and contributed to the longitudinal prediction model. IL-8, IL-10, and GCSF, along with IL-17A, also contributed to the random forest prediction model of Hoehn and Yahr staging. That there is not a perfect overlap between the cytokines contributing to the prediction models of UPDRS III and Hoehn and Yahr may reflect the intricacies of the models themselves, or that as a result of being regulated by similar transcription factors, a number of cytokines are highly correlated with each other and can readily substitute in different models. It should also be noted that only the top ten variables are listed for the prediction models and other cytokines may well have made a lesser but the nonetheless important contribution to model performance.

Over the time frame studied we also observed significant progression in scores on the geriatric depression scale. As many as 14 of the 27 cytokines assayed positively correlated with longitudinal changes in the depression scale. This included IL-6, which was the main contributing cytokine to performance of the random forest prediction model. Serum and CSF levels of IL-6 have previously been linked to depression and mortality in PD,^45–47^ and our results further suggest that underlying inflammatory phenotypes may contribute to a greater risk of progression to depression in PD patients.

Models that allow for better clinical trial design and/or patient stratification would be advantageous; however, further refinements of the prediction models from the current study are likely required before they achieve clinical utility. For all prediction models the main contributing variable was the baseline value for the specific clinical scale being predicted. This is likely due to the limited progression and/or limited sensitivity of the measurement scales of the clinical symptoms that occurred over 2 years. Although progression on the motor severity scales was modest and unlikely to be personally meaningful to patients, predicting longitudinal outcomes over short duration time frames in established PD populations may have utility in clinical trials. For example the recent exenatide trial for PD used a similar cohort of patients (~8 years duration), a similar short time frame (60 weeks) and showed a similar rate of progression over time (2.1 point change in UPDRS).^48^ Thus, prediction models of progression may allow for better clinical trial outcomes by either enriching for subjects most likely to progress the most over a trial time frame, and/or allowing responses to medications to be evaluated on a personal level by comparing individual outcomes to what was predicted for that patient. Replication of our results with a larger sample size and across more diverse ethnic cohorts will be important. It will also be important in subsequent studies to address caveats in our current study that include a lack of information on potential medication use and its influence on clinical scale outcomes or cytokine measures. Cohorts of Ashkenazi Jewish descent are also likely to be enriched in glucocerebrosidase mutations,^49^ which may also influence PD progression, particularly in regard to early motor progression^50^ and later cognitive decline.^51^ Indeed, a similar machine learning approach to that used in our study has demonstrated that diverse genetic polymorphisms can also predict longitudinal clinical outcomes.^39^ Moreover, other factors outside of peripheral inflammation and genetics very likely contribute to PD progression. Indeed, Mollenhauer et al. have demonstrated that diverse factors such as glucose levels, hypertension, uric acid, and cholesterol also contributed to the longitudinal prediction of PD progression.^52^ Thus, our results suggest that peripheral cytokines may contribute to predictive models of PD progression and serve as such a starting point for further measures to be added and evaluated for their performance to predict PD progression in a quantitative manner. The development of better prediction models may contribute to substantially improved clinical trial outcomes, potentially including new therapies such as LRRK2 inhibitors. Future studies could also include prodromal or at risk carrier groups to determine if peripheral cytokines also have utility in predicting symptomology more associated with PD risk, and that did not significantly progress in our established PD cohort, such as olfaction and sleep dysfunction.

## Methods

### Patient samples and clinical data

Serum samples and corresponding clinical data were obtained from the Michael J Fox Foundation LRRK2 cohort consortium (LCC). For further and up-to-date information on the study visit https://www.michaeljfox.org/page.html?lrrk2-cohort-consortium. The LRRK2 clinical cohort consortium collection comprises two studies, a LRRK2 cross-sectional study with samples contributed from 17 countries, and a longitudinal study of an Ashkenazi Jewish population. We have previously analyzed inflammatory cytokine profiles in the cross-sectional study serum samples and this data has been published.^36^ In the current study we have obtained serum samples from an additional 160 Parkinson’s patients from the longitudinal study. Of these patients, 80 have the LRRK2 G2019S mutation. We also obtained an additional 126 serum samples from the same patients that were collected at a 1-year longitudinal follow up, and additional clinical data from 76 of the same patients at a 2-year longitudinal follow up (Fig. 1a). The study was approved by the University of Sydney Human Research Ethics Committee (2017/076). All cohort consortium samples were obtained with informed consent. Inflammatory cytokines were assayed in the serum samples as outlined below. Detailed information on serum sample and patient data collection is available from the LRRK2 clinical cohort consortium website. The following exclusion criteria were applied during sample selection: history of repeated head injury, encephalitis, cerebral tumor, MPTP exposure, stroke, epilepsy, inflammatory diseases of the brain, and skull fractures. Serum samples were shipped to Australia on dry ice and stored at −80 °C until analysis. All inflammatory cytokine assays were performed blinded. The following matching clinical data were also obtained from the LCC where available: age, gender, date of diagnosis, disease duration, UPDRSIII, Montreal cognitive assessment, Epworth sleep scale, Geriatric depression scale, Hoehn and Yahr, UPSIT, REM-sleep disorder scale, the SCOPA-AUT autonomic dysfunction scale, and the modified Schwab and England activities of daily living scale. Baseline demographic and clinical data are shown in Table 1.

### Inflammatory cytokine assays

A magnetic human cytokine multiplex assay (Biorad) was used to simultaneously measure 27 cytokines (MIP1β, IL-6, IFNγ, IL-1RA, IL-5, GM-CSF, TNFα, CCL5, IL-2, IL-1β, CCL11, bFGF, VEGF, PDGF, CXCL10, IL-13, IL-4, MCP-1, IL-8, MIP1α, IL-10, GCSF, IL-15, IL-7, IL-12(p70), IL-17A, and IL-9) in the serum samples. The bioplex assay was performed exactly as per the manufacturers’ instructions, using the recommended one in four dilution of serum, and plates analyzed using a Magpix plate reader (Luminex). Enclosed standards were used to generate an eight-point standard curve to which a five-parameter logistic curve was fitted and used to quantify unknown cytokine concentrations using the Xponent software package (Luminex). Reference pool standards were included in every plate. The coefficient of variance between duplicates was generally <10%. The coefficient of variance between reference pool standards run on separate plates was ~10–40%, depending on the cytokine of interest. Once the ELISA assays were completed, the raw data were uploaded to the Michael J Fox Foundation LCC website and the investigators were then unblinded to facilitate analysis.

### Statistics and data analysis

Statistics and data analysis were performed using SPSS or R with significance set at *p* < 0.05. Missing cases were excluded for all analyses. Student’s *t* test or univariate analysis covarying for age and gender were used to compare between two groups where indicated. Repeated measures analysis of variance was used to analyze longitudinal clinical data. The Shapiro–Wilk test was used to assess normal distribution. The Kolmogorov–Smirnov test was used to compare the distribution of longitudinal changes in variables between the two groups. Pearson and Spearman’s rank correlations were performed to assess relationships between cytokine and clinical variables. For predictive modeling with machine learning, the elastic-net^53^ and random forest^54^ algorithms were trained on combinations of clinical variables, inflammatory cytokine measurements, and demographic variables (age and gender) to predict 2-year longitudinal clinical outcomes. Elastic net is a linear regression technique that applies both L1 and L2 regularizations in order to minimize overfitting. In L1-regularization, the absolute values of the coefficients were used as the penalty term in the loss function, whereas in L2-regularization, the squared values of the coefficients were used. The hyperparameters *λ* and *α* control the strength of the regularization and the ratio between L1 and L2, respectively. Random forest is an ensemble learning algorithm based on multiple decision trees and bootstrap aggregation. For each tree, a bootstrap of samples was used and, at each node of each tree, the best variable out of a random subset of *mtry* variables was selected to be used as the node. Random forest analysis can capture nonlinear relationships and variable importance can be ascertained by the increase in mean squared error after a variable is permuted, with large error increases expected for important variables. For both algorithms the dataset was randomly split such that 80% of the samples were used for training and 20% were withheld for final validation. For each cytokine variable, samples where the log2 ratio between baseline and 1-year follow up were more than two standard deviations away from the cohort mean were removed, as these likely reflect extraneous underlying inflammatory conditions. For log2 ratio calculation, a pseudocount of one was added to the clinical markers for the log2 ratio calculation to avoid log(0) scenarios. Tenfold cross-validation was performed on the training set to identify the *λ* and *α* values for elastic net, and *mtry* values for random forest, that best minimized the root mean squared error (RMSE). Elastic-net and random forest (500 trees) models were then constructed on the whole training set with these optimal hyperparameter values. Generalizability was tested on the withheld validation set and assessed with performance metrics RMSE and NRMSE, which was determined by dividing the RMSE by the range of possible values for that clinical specific marker. An NRMSE score of zero is equivalent to a prediction model that operates with 100% accuracy.

### Reporting summary

Further information on research design is available in the Nature Research Reporting Summary linked to this article.

## Supplementary information

Supplemental Material

Reporting Summary

## Data Availability

The inflammatory cytokine and clinical data used in this paper are available for download from the LRRK2 clinical consortium website, subject to approval from the Michael J Fox Foundation.

## Supplementary information

**Supplementary information** accompanies the paper on the *npj Parkinson’s Disease* website (10.1038/s41531-019-0086-4).

## Code availability

Code used to analyze the datasets is available from the corresponding author upon reasonable request.
