# Supplementary material for: Parkinson’s progression prediction using machine learning and serum cytokines
Source: NPJ Parkinsons Dis. 2019 Jul 25;5:14. doi: 10.1038/s41531-019-0086-4 (PMC6658482; doi:10.1038/s41531-019-0086-4)
Supplement: Supplementary file 1 — Supplemental Material [file 41531_2019_86_MOESM1_ESM.pdf]

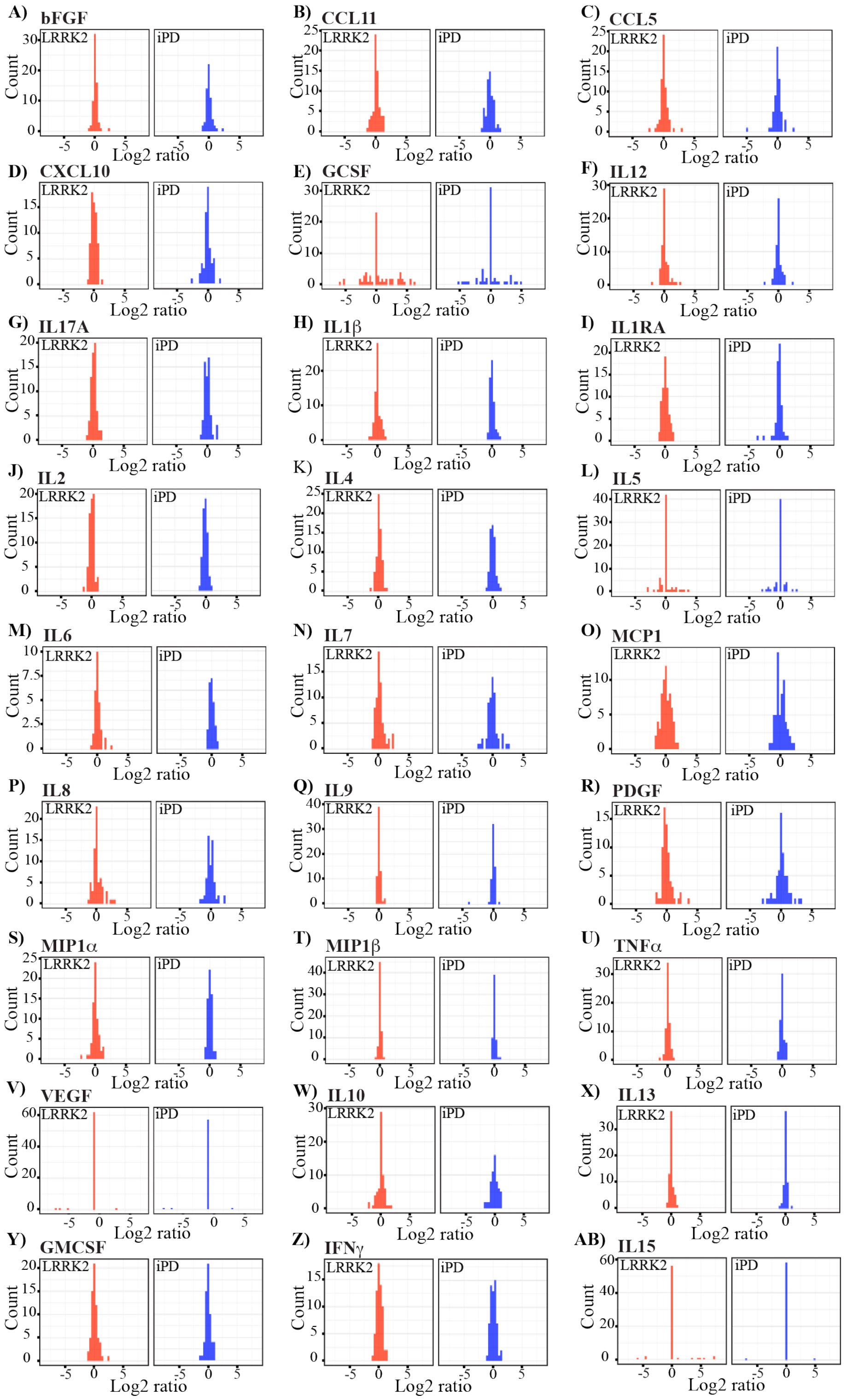

Supplementary Figure 1. Changes in individual cytokine levels over 1 year. The change in protein levels between baseline and year 1 was calculated for each of the 27 cytokines assayed and for both the LRRK2 (red) and idiopathic (blue) Parkinson's disease groups. On the graphs the x-axis indicates the log2 fold change and the y axis indicates the number of measures for each particular fold change.

Cytokine	Idiopathic PD	LRRK2 PD
MIP1β	143.58 ± 2.52	148.34 ± 3.47
IL6	4.14 ± 2.06	2.31 ± 0.11
IL1RA	140.97 ± 9.42	151.11 ± 8.96
IL5	7.70 ± 6.95	0.86 ± 0.20
TNFα	42.31 ± 3.01	40.68 ± 1.40
CCL5	13495.28 ± 464.74	13784.25 ± 478.57
IL2	4.93 ± 1.16	3.75 ± 0.13
IL1β	2.66 ± 1.28	1.47 ± 0.11
CCL11	105.59 ± 4.89	106.95 ± 5.58
bFGF	44.06 ± 7.42	37.65 ± 1.18
VEGF	3.57 ± 2.23	1.64 ± 1.02
PDGF	3636.89 ± 191.48	4484.02 ± 207.95*
CXCL10	739.22 ± 54.08	719.95 ± 40.63
IL13	0.72 ± 0.05	0.69 ± 0.05
IL4	2.97 ± 0.13	3.06 ± 0.13
MCP1	23 ± 1.27	29.24 ± 2.04*
IL8	10.47 ± 0.70	10.63 ± 0.48
MIP1α	2.11 ± 0.10	2.58 ± 0.20
IL10	1.21 ± 0.12	1.57 ± 0.27
GCSF	9.97 ± 4.99	9.38 ± 1.94
IL7	4.54 ± 0.25	4.85 ± 0.25
IL12	10.12 ± 8.03	2.59 ± 0.44
IL17A	13.26 ± 0.50	13.61 ± 0.43
IL9	99.51 ± 3.71	97.86 ± 2.08
GMCSF	0.88 ± 0.15	0.79 ± 0.07
IFNγ	2.79 ± 0.18	2.83 ± 0.17
IL15	2.11 ± 1.69	1.47 ± 0.84

Supplementary Table 1. Serum cytokine levels in idiopathic and LRRK2-PD. Multiplex ELISA assay was used to measure the protein levels of 27 cytokines at baseline in serum from Parkinson's disease patients with and without the LRRK2 G2019S mutation. Data are mean ± SEM, n=80 per group. * = $p < 0.05$ using Student's T-test.

	Spearman's rho	Sig. (2-tailed)
Epworth Sleep Scale	0.0	0.998
Geriatric Depression Scale	-0.113	0.240
Hoehn and Yahr	-0.057	0.552
Schwab and England ADL	0.065	0.506
SCOPA-AUT	-0.051	0.577
REM sleep dysfunction	-0.039	0.677
MoCA	-0.001	0.992
UPSIT	0.096	0.291
UPDRS III	-0.062	0.493

Supplementary Table 2. Lack of correlation between serum PDGF and PD symptoms. Spearman's rank correlations were performed to determine if serum PDGF significantly associated with clinical variables in the PD cohort. Data are mean ± SEM, n=160.

	Random Forest			Elastic Net			
**Clinical Variables**	**Mtry**	**Training RMSE**	**Training NRMSE**	**Alpha**	**Lambda**	**Training RMSE**	**Training NRMSE**
Geriatric Depression Scale	28	1.0301	0.0687	0.7	0.881	3.074	0.2049
Hoehn and Yahr	25	0.2178	0.0544	0.7	0.114	0.3795	0.0949
Schwab and England ADL	28	4.0746	0.0582	1	3.9	13.6077	0.1944
UPDRS III	28	4.109	0.0596	1	1.872	12.3792	0.1768

Supplementary Table 3. Hyperparameters and training performance for machine learning algorithms. The *mtry* hyperparameter controls the number of variables randomly selected for splitting in random forest. The α and λ hyperparameters control the regularisation of the elastic-net algorithm. The optimal value for all hyperparameters were identified with 10-fold cross-validation on the training set. The root mean square error (RMSE) is a measure of performance on the training data set. The normalized root mean square error (NRMSE) controls for differences in the range of the clinical scales and allows cross comparison. For both RMSE and NRMSE, the lower the value the better the model has performed.

	Random Forest Clinical data only			Random Forest Clinical and cytokine data		
**Clinical Variables**	**Mtry**	**Training NRMSE**	**Test NRMSE**	**Mtry**	**Training NRMSE**	**Test NRMSE**
Geriatric Depression Scale	12	0.0727	0.1914	31	0.0667	0.1955
Hoehn and Yahr	25	0.0496	0.1397	0.7	0.0506	0.1148
Schwab and England ADL	2	0.0644	0.206	1	0.583	0.2337
UPDRS III	8	0.0556	0.1431	1	0.577	0.1312

Supplementary Table 4. Training and test performance for random forest prediction models. Random forest was used to predict three-year longitudinal clinical outcomes using just available clinical variables or the clinical variables combined with baseline cytokines. The *mtry* hyperparameter controls the number of variables randomly selected for splitting in random forest. The optimal value for all hyperparameters were identified with 10-fold cross-validation on the training set. The normalized root mean square error (NRMSE) controls for differences in the range of the clinical scales and allows cross comparison. For NRMSE, the lower the value the better the model has performed.
