# Supplementary material for: Parkinson’s progression prediction using machine learning and serum cytokines
Source: NPJ Parkinsons Dis. 2019 Jul 25;5:14. doi: 10.1038/s41531-019-0086-4 (PMC6658482; doi:10.1038/s41531-019-0086-4)
Supplement: Supplementary file 2 — Reporting Summary [file 41531_2019_86_MOESM2_ESM.pdf]

nature research

Corresponding author(s): Nicolas Dzamko

Last updated by author(s): May 17, 2019

Reporting Summary

Nature Research wishes to improve the reproducibility of the work that we publish. This form provides structure for consistency and transparency in reporting. For further information on Nature Research policies, see Authors & Referees and the Editorial Policy Checklist.

Statistics

For all statistical analyses, confirm that the following items are present in the figure legend, table legend, main text, or Methods section.

n/a	Confirmed	
☐	☒	The exact sample size (n) for each experimental group/condition, given as a discrete number and unit of measurement
☐	☒	A statement on whether measurements were taken from distinct samples or whether the same sample was measured repeatedly
☐	☒	The statistical test(s) used AND whether they are one- or two-sided<br>*Only common tests should be described solely by name; describe more complex techniques in the Methods section.*
☐	☒	A description of all covariates tested
☐	☒	A description of any assumptions or corrections, such as tests of normality and adjustment for multiple comparisons
☐	☒	A full description of the statistical parameters including central tendency (e.g. means) or other basic estimates (e.g. regression coefficient) AND variation (e.g. standard deviation) or associated estimates of uncertainty (e.g. confidence intervals)
☐	☒	For null hypothesis testing, the test statistic (e.g. F, t, r) with confidence intervals, effect sizes, degrees of freedom and P value noted<br>*Give P values as exact values whenever suitable.*
☒	☐	For Bayesian analysis, information on the choice of priors and Markov chain Monte Carlo settings
☐	☒	For hierarchical and complex designs, identification of the appropriate level for tests and full reporting of outcomes
☐	☒	Estimates of effect sizes (e.g. Cohen's d, Pearson's r), indicating how they were calculated

Our web collection on statistics for biologists contains articles on many of the points above.

Software and code

Policy information about availability of computer code

Data collection	Data collection used Bioplex software (ver 6) for cytokine measurements.
Data analysis	Data analysis used microsoft excel, graphpad prism (ver 7), SPSS (ver 24) and R (ver 3)

For manuscripts utilizing custom algorithms or software that are central to the research but not yet described in published literature, software must be made available to editors/reviewers. We strongly encourage code deposition in a community repository (e.g. GitHub). See the Nature Research guidelines for submitting code & software for further information.

Data

Policy information about availability of data

All manuscripts must include a data availability statement. This statement should provide the following information, where applicable:

- Accession codes, unique identifiers, or web links for publicly available datasets
- A list of figures that have associated raw data
- A description of any restrictions on data availability

The inflammatory cytokine and clinical data are available for download from the LRRK2 clinical consortium website, subject to approval from the Michael J Fox Foundation.

Field-specific reporting

Please select the one below that is the best fit for your research. If you are not sure, read the appropriate sections before making your selection.

☒ Life sciences ☐ Behavioural & social sciences ☐ Ecological, evolutionary & environmental sciences

Life sciences study design

All studies must disclose on these points even when the disclosure is negative.

Sample size	Sample size was based on the availability of the longitudinally collected samples.
Data exclusions	Cases where data was missing were excluded, rather than imputing missing clinical data. Cases were excluded from the machine learning analysis if they had a 1 year change in cytokine levels greater than 2 standard deviations from baseline.
Replication	Aspects of this study did replicate our previous published work. For new analyses replication will need to be performed once additional cohorts have been identified to facilitate this.
Randomization	Patients were assigned to groups based on the diagnosis of Parkinson's disease and the presence / absence of a LRRK2 G2019S mutation
Blinding	Cytokine measurements and data collection were performed blinded. Only after returning the raw data set to the LRRK2 consortium were investigators unblinded.

Reporting for specific materials, systems and methods

We require information from authors about some types of materials, experimental systems and methods used in many studies. Here, indicate whether each material, system or method listed is relevant to your study. If you are not sure if a list item applies to your research, read the appropriate section before selecting a response.

Materials & experimental systems

n/a	Involved in the study	
☒	☐	Antibodies
☒	☐	Eukaryotic cell lines
☒	☐	Palaeontology
☒	☐	Animals and other organisms
☐	☒	Human research participants
☒	☐	Clinical data

Methods

n/a	Involved in the study	
☒	☐	ChIP-seq
☒	☐	Flow cytometry
☒	☐	MRI-based neuroimaging

Human research participants

Policy information about studies involving human research participants

Population characteristics	Demographic data was available to covary for age, gender and LRRK2 mutation status. All patients had a confirmed diagnosis of Parkinson's disease.
Recruitment	Patients were recruited through the LRRK2 clinical consortium. Our study applies a retrospective analysis of samples previously collected. We did not recruit any patients ourselves for this study.
Ethics oversight	The University of Sydney human research ethics committee

Note that full information on the approval of the study protocol must also be provided in the manuscript.
